# Insight into the mechanisms and dysregulation of KMT5C-H4K20me3 in cancer

**DOI:** 10.1080/15592294.2025.2574007

**Published:** 2025-10-17

**Authors:** Jihye Son, Andrea L. Kasinski

**Affiliations:** a Department of Biological Sciences, Purdue University, West Lafayette, IN, USA; b Purdue Institute for Cancer Research, Purdue University, West Lafayette, IN, USA

**Keywords:** KMT5C, SUV420H2, H4K20me3, epigenetics, cancer

## Abstract

KMT5C-mediated histone H4 lysine 20 trimethylation (H4K20me3) has traditionally been linked to heterochromatin formation and maintenance, playing a crucial role in maintaining genome integrity. Emerging evidence, however, indicates that perturbations of KMT5C-H4K20me3 are also implicated in various cancers, positioning KMT5C-H4K20me3 as a promising target for anti-cancer therapies. Despite this, the precise mechanisms underlying KMT5C recruitment to its genomic targets and the specific genes it regulates remain poorly understood. In this review, we explore the dysregulation of KMT5C-mediated H4K20me3 in cancer, providing a comprehensive overview of its known functions. We also highlight recent findings that suggest a novel, non-canonical pathway for H4K20me3 deposition by KMT5C, and, while early on, insight into future opportunities for therapeutic intervention.

## Background

Histone post-translational modifications (PTMs) are critical regulators of gene expression and chromatin dynamics. These covalent modifications influence chromatin structure and function by directly altering nucleosome compaction or by serving as docking sites for chromatin-associated proteins. Such changes modulate DNA accessibility to the transcriptional machinery, ultimately shaping gene expression profiles. Among the diverse PTMs, acetylation and methylation are two of the most extensively studied and functionally significant marks [1–3].

Acetylation occurs on lysine residues and neutralizes their positive charge, directly leading to chromatin decompaction and enhanced DNA accessibility – features typically associated with transcriptional activation. In contrast, histone methylation does not alter charge and therefore does not *directly* affect histone-DNA interactions. Instead, methylated histones serve as docking sites for secondary factors that can ‘read,’ ‘write,’ or ‘erase’ PTMs, or for downstream effector complexes. The outcome of histone methylation depends heavily on the specific site and degree of methylation – whether mono-, di-, or tri-methylation on lysine residues, or mono-, or di-methylation on arginine residues [3,4].

Due to the combinatorial diversity of methylation patterns and their frequent crosstalk with other histone PTMs, these marks orchestrate distinct transcriptional programs. For example, trimethylation of lysine 9 on histone H3 (H3K9me3) is associated with transcriptionally inactive heterochromatin [5], whereas trimethylation of lysine 4 on histone H3 (H3K4me3) is typically enriched at active gene promotors in euchromatin [6]. Thus, it is perhaps not surprising that dysregulation of genes encoding chromatin-modifying enzymes, including those that regulate histone methylation, is found in 10–20% of all cancers [4], reinforcing the concept of epigenetic reprogramming as a novel emerging hallmark of cancer [7]. More specifically, alterations in histone modification patterns – including aberrant histone methylation – are now recognized as key contributors to tumor heterogeneity [8].

Several examples illustrate the importance of histone methylation in cancer development. Transient loss of the Polycomb Repressive Complex (PRC), which deposits H3K27me3, can lead to heritable changes in chromatin accessibility and initiate tumorigenesis [9]. NSD3, a methyltransferase that deposits H3K36me3, is frequently amplified in lung squamous cell carcinoma (LUSC), and genetic ablation of NSD3 attenuates tumor growth and extends survival of LUSC mouse models [10]. In pediatric high-grade gliomas (pHGGs), a lysine-to-methionine mutation at position 27 in histone H3.3 (H3.3K27M) occurs in approximately 50% of cases and leads to a global reduction in H3K27me3 levels [11]. This epigenetic shift alters transcriptional programs and is believed to contribute to tumor initiation and progression [12].

Given the pivotal role of histone PTMs in cancer biology, there is growing interest in targeting epigenetic regulators therapeutically. For instance, several histone deacetylase (HDAC) inhibitors, including vorinostat, romidepsin, and belinostat, have received US Food and Drug Administration (FDA) approval for treating T cell lymphomas [13]. Likewise, inhibitors targeting histone methyltransferases are being explored. KTX-1001, an inhibitor of Multiple Myeloma SET domain (MMSET, also known as NSD2), is currently in clinical trial for multiple myeloma patients with MMSET overexpression (ClinicalTrials.gov ID NCT05651932). Furthermore, an inhibitor of Enhancer of Zeste Homolog 2 (EZH2), the catalytic subunit of the PRC, has been FDA approved for the treatment of follicular lymphoma and epithelioid sarcoma [14,15].

Despite these advances, a major challenge with epigenetic therapies is off-target toxicity due to the global role of chromatin modifiers in regulating gene expression [16]. These enzymes often have non-histone substrates as well, further complicating therapeutic specificity. This has spurred interest in developing locus-specific epigenetic strategies. For example, adeno-associated virus (AAV)-mediated delivery of DNMT3A successfully induces DNA methylation and silencing of *PRNP* in the brains of mice with prion disease [17]. This proof-of-concept opens the door to potential applications in cancer, where targeted activation of tumor-suppressive genes or silencing of oncogenes through epigenetic mechanisms may be used to reduce tumor burden and overcome drug resistance mechanisms.

Nonetheless, whether epigenetic therapies act globally or locally, a thorough understanding of their mechanisms of action, gene targets, and context-specific effects is essential to minimize toxicity and maximize therapeutic benefit.

In this review, we focus on a relatively understudied histone PTM – trimethylation of histone H4 at lysine 20 (H4K20me3) – which is dysregulated in multiple cancers (Table 1). Although H4K20me3 was first identified in the 1960s and the mechanisms of its deposition characterized over two decades ago [18], its functional roles in transcription regulation and tumor progression remain incompletely understood. Here, we review the literature surrounding the dysregulation of H4K20me3 in cancer and its potential as a therapeutic target. We also examine canonical and emerging non-canonical mechanisms of H4K20me3 deposition, with an eye toward future development of epigenetic-mediated anti-cancer therapeutics.

**Table 1.** Summary of alteration of KMT5C-H4K20me3 levels across cancers.

Cancer type	KMT5C-H4K20me3 levels	Reference
Bone	Down	[57]
Breast	Down	[54–56]
Colon	Down	[59,60]
Leukemia	Down	[60]
Lung	Down	[24,58,60]
Lymphoma	Down	[60]
Oral	Down	[61]
Skin	Down	[60]
Kidney	Up	[62,63]
Liver	Up	[64]
Pancreas	Up	[25]

## KMT5C-H4K20me3 activity and regulation of chromatin

### KMT5 family members coordinate sequential methylation of H4K20

**Figure 1.**
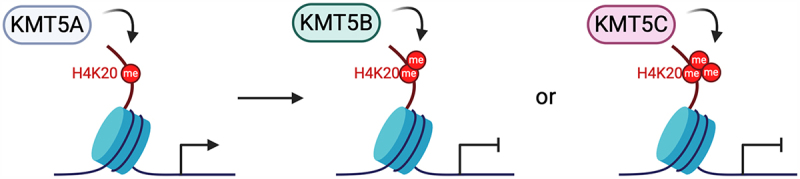
Lysine methyltransferases responsible for deposition of methylation on histone H4 lysine 20 (H4K20): KMT5A (also known as SETD8, or SET8) catalyzes H4K20me1 using H4K20 as its substrate. KMT5B and KMT5C both utilize H4K20me1 as their substrate and catalyze H4K20me2 and H4K20me3, respectively.

The methylation of lysine residues on histones is catalyzed by lysine methyltransferases (KMT), which are highly specific in substrate recognition and modification. In the case of H4K20me3, unmethylated H4K20 is initially monomethylated by KMT5A (also known as SETD8 or SET8) to generate H4K20me1. This monomethylated intermediate then serves as a substrate for either KMT5B or KMT5C, which catalyze further methylation to form H4K20me2 and H4K20me3, respectively [18,19] (Figure 1).

### KMT5B and KMT5C have distinct roles in H4K20 methylation in vivo

Although KMT5B and KMT5C share significant sequence and structural similarity, *in vivo* studies reveal that they play non-redundant roles in H4K20 methylation [20,21]. *In vitro* assays show that both enzymes can utilize H4K20me1 as a substrate to generate H4K20me2 [19,22]. However, only KMT5C has been shown to catalyze the formation of H4K20me3 *in vivo*. Specifically, KMT5C-deficient mouse embryonic fibroblasts (MEFs) exhibit a marked reduction in H4K20me3, with H4K20me2 levels remaining unaffected. In contrast, KMT5B-deficinet MEFs show a significant decrease in H4K20me2, but not H4K20me3 [23]. These findings support a division of labor *in vivo*, where KMT5B is primarily responsible for generating H4K20me2 and KMT5C is essential for H4K20me3. Intriguingly, these data also suggest that KMT5C may require an additional, as-yet unidentified co-factor to achieve full catalytic activity in cells.

### KMT5C is the major methyltransferase for H4K20me3 across tissues and cells

Multiple independent studies further support the central role of KMT5C as the major methyltransferase responsible for H4K20me3. In lung cancer cell lines (EKVX, HCC827, and PC9) engineered to express catalytically inactive KMT5C mutants, H4K20me3 levels were significantly reduced [24]. Similarly, siRNA-mediated knockdown of KMT5C in pancreatic cancer cells (PANC1, Hs766T, and KP4) and brown adipocytes cells (BAT1) led to global loss of H4K20me3 [25,26], while, knockdown of KMT5C in adipocytes strongly diminished H4K20me3 deposition [27]. Despite this reduction, H4K20me3 was not entirely eliminated, suggesting that KMT5B may partially compensate to maintain basal levels of trimethylation, or another, unidentified methyltransferase may be involved. Some studies have proposed that SMYD3, a known H3K4me3 methyltransferase, might also catalyze H4K20me3 [28–32], but these findings are not unanimous as other groups have reported conflicting data [33,34], highlighting the need for further studies to clarify the function of SMYD3 in generating H4K20me3. Likewise, SMYD5 has been suggested to catalyze H4K20me3 [35], but this finding has also been challenged [36,37]. Altogether, current evidence strongly supports KMT5C as the bona fide primary enzyme responsible for H4K20me3 deposition in cells and tissues.

### KMT5C-mediated H4K20me3 functions in both constitutive and facultative heterochromatin

KMT5C-mediated H4K20me3 has traditionally been associated with constitutive heterochromatin, particularly within pericentric and telomeric regions [18,38]. KMT5C interacts with heterochromatin protein 1 (HP1) and co-localizes with repressive marks such as H3K9me3 and DAPI staining in condensed chromatin [18,39]. Fluorescence recovery after photobleaching (FRAP) experiments demonstrate that KMT5C is stably bound to these heterochromatic regions marked by dense Hoechst staining [39]. However, emerging studies have shown that KMT5C-H4K20me3 is not confined to gene-poor, repressive chromatin. It also localizes within gene-rich euchromatic regions to mediate transcriptional repression. For example, in lung cancer cells, KMT5C deposits H4K20me3 at the promoter of the long non-coding RNA, *LINC01510*, repressing its expression [24]. In breast cancer cells, KMT5C facilitates RNA polymerase II pausing at the *TMS1* promoter through H4K20me3 [40]. Additionally, H4K20me3 deposition at the promoter of ribosomal DNA (rDNA) promotes local chromatin compaction [41]. These findings expand the functional repertoire of KMT5C-H4K20me3 and highlight its role in transcriptional regulation of specific genomic loci.

Despite these insights into KMT5C activity in euchromatic regions, the full extent of H4K20me3 deposition in gene-rich areas remains poorly defined. Most studies to date have focused on its role in constitutive heterochromatin, leaving a gap in understanding how H4K20me3 is distributed across active chromatin landscapes. This lack of comprehensive localization data underscores the need for genome-wide epigenomic and transcriptomic analyses to identify target genes directly regulated by KMT5C-H4K20me3. Such studies will be critical for fully elucidating the role of KMT5C-H4K20me3 in transcriptional repression and for determining whether its euchromatic functions are context-specific or broadly conserved across cell types and cancer subtypes.

## Canonical mechanism of H4K20me3 deposition by KMT5C

**Figure 2.**
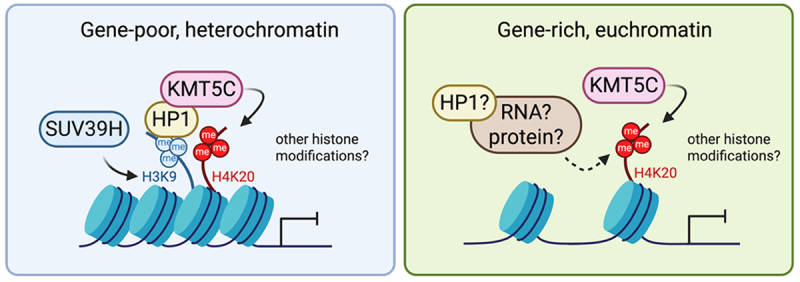
Sequential deposition of H4K20me3 by KMT5C in gene-poor, heterochromatin: HP1 binds to H3K9me3 (catalyzed by SUV39H) and recruits KMT5C to chromatin. KMT5C utilizes H4K20me1, which was established by KMT5A, and generates H4K20me3. It has been suggested that HP1-inpendendent KMT5C recruitment pathways exist in gene-rich euchromatin, but this model requires further characterization. It would also be essential to investigate how H4K20me3 engages in crosstalk with other histone modifications to orchestrate the effect of H4K20me3.

To harness KMT5C-mediated H4K20me3 as a therapeutic modality, it is critical to define its mechanism of action and the gene networks it regulates. Thus far, H4K20me3 deposition by KMT5C has been well characterized in constitutive heterochromatin, including pericentric and telomeric regions. In these domains, deposition occurs sequentially (Figure 2, left): SUV39H first catalyzes H3K9me3, which is recognized by the chromodomain of HP1. HP1 then recruits KMT5C, which converts H4K20me1 to H4K20me3, as outlined in Figure 1 [18,19,22].

This HP1-dependent deposition mechanism, first identified in *Drosophila*, is conserved across multiple model systems, including human cells and tissues [18,41,42]. Mammalian cells express three HP1 isoforms (HP1⍺, HP1β, and HP1ɣ). Although KMT5C can bind all three, HP1β is often cited as the primary mediator of KMT5C recruitment [43]. However, other studies suggest HP1ɣ is also critical, as its loss reduces H4K20me3 levels [44,45]. Whether these isoform-specific functions are conserved across tissues, or whether redundancy allows compensation when one isoform is disrupted, remains unresolved. Nonetheless, HP1 is well established as the key factor sequestering KMT5C at H3K9me3-marked heterochromatin, where it remains stably bound [39].

Consistent with H3K9me3 acting upstream of H4K20me3, epigenomic profiling of the National Cancer Institute (NCI)-60 cancer cell lines demonstrates strong colocalization of these marks across heterochromatic regions [46]. Yet, cytogenetic banding patterns of H4K20me3 do not fully overlap with those of H3K9me3 [46]. While most H4K20me3 is enriched in compact heterochromatin, a subset is also detected in gene-rich euchromatic regions [46]. This suggests that H4K20me3 may promote transient formation of facultative heterochromatin, leading to local transcriptional repression. The presence of H4K20me3 at sites lacking H3K9me3 further implies alternative deposition pathways – potentially via HP1-independent KMT5C recruitment or through an additional, unidentified methyltransferase. Whether KMT5C recruitment to euchromatin is HP1-dependent, as in constitutive heterochromatin, or HP1-independent remains unknown and requires further investigation.

## Non-canonical KMT5C-H4K20me3 regulation: HP1-independent recruitment of KMT5C to chromatin

In addition to profiling studies that revealed H4K20me3 deposition at sites lacking H3K9me3 [46], several lines of evidence suggest that HP1 is not the only factor responsible for recruiting KMT5C to chromatin. In one study, MEFs overexpressing either full-length EGFP-tagged KMT5C or a truncated mutant lacking the HP1 interaction domain both showed increased global H4K20me3 levels, though the full-length KMT5C construct produced a stronger effect [47]. These results reinforce the central role of HP1-mediated recruitment, while also indicating that KMT5C can localize to chromatin independently of HP1, likely through additional interacting partners (Figure 2, right).

Consistent with this idea, an independent study demonstrated that KMT5C interacts with the long non-coding RNA, *PAPAS*, which recruits KMT5C to a ribosomal RNA (rRNA) locus in euchromatin. At these sites, KMT5C catalyzes H4K20me3 deposition, promoting chromatin compaction and transcriptional repression. Supporting this model, a KMT5C deficient in HP1 binding retained its interaction with *PAPAS* and its ability to repress rRNA transcription [41]. These findings establish that KMT5C can be recruited through HP1-independent pathways.

Together these observations highlight a non-canonical mode of KMT5C recruitment that likely involves interactions with diverse biomolecules, including proteins and RNAs, to regulate H4K20me3 in transcriptionally active regions. We hypothesize that such HP1-independent pathways may play a critical role in modulating oncogene and tumor suppressor expression through KMT5C-H4K20me3, with important implications for cancer biology. Future studies should aim to identify additional KMT5C-interacting partners beyond HP1 to fully elucidate its role in context-specific gene regulation during homeostasis, stress responses, and disease.

## Dysregulation of KMT5C-H4K20me3 in cancer and potential for therapeutic intervention

### Genomic alterations and post-transcriptional regulation of KMT5C

Single nucleotide polymorphisms (SNPs), which occur in < 1% of the population, have emerged as a potential mechanism influencing chromatin-modifying enzymes, including histone lysine methyltransferases [48]. Notably, a recent study reported that androgens induce SNPs in a locus surrounding *KMT5C* in breast cancer patients. Particularly, a homozygous SNP within this locus was associated with reduced expression of nearby genes, including *KMT5C* [49].

Beyond this example, more than 6,000 SNPs have been catalogued in *KMT5C* in the NIH dbSNP database (https://www.ncbi.nlm.nih.gov/snp/), the vast majority of which remain unexplored. This abundance raises the possibility that genetic variation could directly modulate the methyltransferase activity of KMT5C or its stability. Supporting this idea, several somatic mutations in *KMT5B* (a closely related enzyme with strong structural and sequence similarity to KMT5C) have been shown to profoundly alter its catalytic function [50]. We therefore speculate that SNPs in *KMT5C* may similarly influence its enzymatic activity, stability, or expression. A deeper understanding of these variants will be critical for assessing the feasibility of targeting KMT5C-H4K20me3 as a therapeutic strategy in cancer.

In addition to genomic changes, differential expression of upstream regulatory elements may further modulate KMT5C activity. For example, microRNA-29a, a direct regulator of *KMT5C*, exhibits aberrant and variable expression across cancer types [51,52]. Additionally, several microRNAs (miRNA) involved in EGFR-tyrosine kinase inhibitor (TKI) resistance are predicted to target *KMT5C* [53]. These observations raise the possibility that restoring or targeting KMT5C-H4K20me3 could serve as a therapeutic strategy – perhaps through inhibiting upstream miRNAs that negatively regulate *KMT5C*, but care must be taken to consider the appropriate biological context and the overall role of KMT5C.

### KMT5C-H4K20me3 is frequently downregulated across multiple cancer types, functioning as a tumor suppressor and a mediator of drug sensitivity

Functionally, genome-wide analysis of H4K20me3 deposition across the NCI-60 cancer cell line panel revealed widespread loss of this mark in cancer cell lines [46], suggesting that H4K20me3 is frequently diminished in malignancies. This trend has been independently validated in numerous studies using both patient-derived tumor tissues and representative cancer cell lines, which consistently report reduced levels of KMT5C and H4K20me3 across a wide range of cancer types [24,54–61] (Table 1).

For example, KMT5C was identified as a tumor-suppressor in estrogen receptor (ER)-positive breast cancer. Patients with low levels of H4K20me3 exhibit significantly shorter overall survival [54,56]. Functionally, KMT5C overexpression in breast cancer cells increases H4K20me3 deposition upstream of the *Tensin-3* gene, which is known to promote cell invasion. This methylation-mediated repression of *Tensin-3* leads to reduced invasiveness, whereas KMT5C knockdown enhances it [54,55]. These findings highlight KMT5C as a negative regulator of tumor progression in breast cancer.

KMT5C downregulation may also be involved in promoting therapeutic resistance in lung cancer. In our own study, KMT5C expression was reduced in tumor samples from EGFR-mutant lung cancer patients following treatment with osimertinib, a third-generation EGFR TKI, compared to treatment-naïve matched tissues (unpublished; data from [65]). Mechanistically, KMT5C loss reduced H4K20me3 enrichment at the promoter of *LINC01510*, an oncogenic long non-coding RNA, resulting in its de-repression and subsequent upregulation of *MET*, a gene involved in a well-established EGFR TKI resistance pathway. Nonetheless, re-expression of *LINC01510* or *MET* in KMT5C-depleted cells did not fully restore TKI sensitivity, suggesting that additional pathways downstream of KMT5C loss contribute to resistance and remain to be identified [24].

### Contextual complexity of KMT5C function in cancer

Despite its tumor-suppressive role in several cancers, KMT5C may act as an oncogene in a context-dependent manner (Table 1). In pancreatic cancer, KMT5C-H4K20me3 is associated with increased cell invasiveness [25,54,55]. In renal cell carcinoma, KMT5C promotes cell proliferation by repressing *DHRS2*, a gene that induces apoptosis [62]. Interestingly, higher KMT5C expression in renal tumors correlates with better overall survival in patients with clear cell renal cell carcinoma [63]. In hepatocellular carcinoma (HCC), environmental stress induces KMT5C expression, facilitating its interaction with RAD51, a key component of the DNA damage repair (DDR) pathway. Inhibition or downregulation of KMT5C disrupts RAD51 function and synergizes with poly(ADP-ribose) polymerase (PARP) inhibitors, further supporting an oncogenic role for KMT5C in this setting [64]. These findings suggest that the functional consequences of KMT5C-H4K20me3 are tissue-specific and may vary depending on the cellular environment and stress response pathways.

Even within the same type of cancer, KMT5C may exhibit opposing roles depending on genetic context and tumor heterogeneity, adding another layer of complexity. For instance, TP53-mutant or null breast and lung cancer cells are especially sensitive to combined inhibition of KMT5C and aurora kinase B (AURKB), which governs chromosome segregation. In contrast, wild-type TP53 protects cells from mitotic catastrophe under dual inhibition, leading to polyploidy and death only in TP53-deficient cells [66]. Additionally, KMT5C-H4K20me3 levels in cancer cells are highly dynamic and responsive to cellular stress, even in the same cell lines. For example, in proliferating cancer cells, such as SK-Mel-147 (melanoma) and A549 (lung), H4K20me3 levels are low, and their survival improves upon co-downregulation of KMT5B and KMT5C, which is consistent with prior findings of KMT5C-H4K20me3 acting as a tumor suppressor in melanoma and lung cancer [24,58,60]. In contrast, senescent or drug-tolerant persister cells accumulate high levels of H4K20me3, and their survival is compromised when KMT5B and KMT5C are co-inhibited [67]. Such context-dependent role of KMT5C may also explain its recently reported oncogenic role in lung cancer [68], highlighting the need for future research to reconcile these conflicting functions of KMT5C. These findings demonstrate that KMT5C-H4K20me3 levels shift dynamically in response to therapeutic and environmental stresses, adding complexity to its role in tumor biology.

The context-dependent function of KMT5C-H4K20me3 may be explained by cell type-specific differences in genomic targets and upstream regulators. Epigenomic profiling of the NCI-60 cancer cell line panel revealed considerable heterogeneity in H4K20me3 deposition, suggesting that KMT5C represses different gene networks depending on tissue of origin [46].

### Towards therapeutic targeting or KMT5C-H4K20me3: next steps

To realize the therapeutic potential of targeting KMT5C-H4K20me3, future studies must define the consequences of genetic and epigenetic alterations in KMT5C, its upstream regulatory mechanisms, and its context-specific functional targets. Systematic efforts to catalog and characterize SNPs and other genomic alterations may clarify whether specific perturbations underlie KMT5C misregulation. Integrating genome-wide epigenomic profiling (i.e., CUT&RUN for KMT5C and H4K20me3) with transcriptomic analysis following KMT5C perturbation could help map the gene programs governed by this methylation mark. In parallel, CRISPR-based functional screens to identify upstream regulators could uncover pathways that control KMT5C expression and activity across distinct cancer types [69]. Such knowledge could provide indirect means to fine-tune KMT5C expression. Collectively, these approaches will be essential to design precision epigenetic therapies that exploit KMT5C-H4K20me3 as a cancer vulnerability while minimizing off-target effects. Although no KMT5C inhibitors have yet reached to the clinic, promising preclinical studies suggest that such agents may be on the horizon.

**Figure 3.**
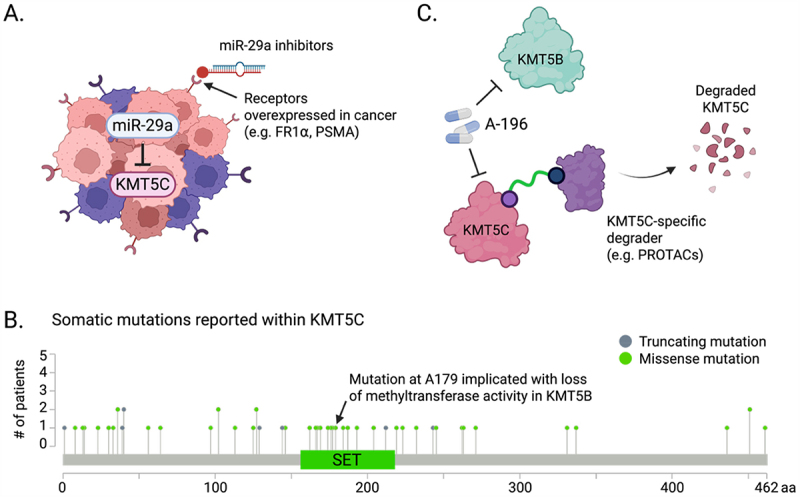
Opportunities for therapeutic intervention targeting KMT5C. A. miRNA-based therapeutics: miR-29a is a direct upstream regulator of KMT5C and its level can be modulated using miR-29a inhibitors. Cancer-specific delivery can be achieved by utilizing receptors that are overexpressed in cancer. B. Somatic mutations reported to occur within KMT5C (data acquired from cBioportal). In particular, mutation in A179 residue has been shown to be implicated in loss of methyltransferase activity in KMT5B, suggesting a similar effect could exist in KMT5C. C. Although A-196 inhibits the methyltransferase activity of KMT5C, KMT5B is also affected, calling for the need to develop KMT5C-specific degraders.

One strategy to restore or downregulate KMT5C-H4K20me3 involves miRNA-based therapeutics. For example, antagonizing miRNAs that inhibit KMT5C, such as miR-29a, which is upregulated in multiple types of cancer, could enhance KMT5C abundance and function [51]. With advances in targeted RNA delivery, including the use of ligands that bind receptors overexpressed in cancer cells, miR-29a inhibitors could be selectively delivered to tumors, thereby reducing toxicity to healthy cells. Chemical modifications of the miR-29a inhibitors could further improve their stability and activity (Figure 3A) [70,71]. Future studies investigating miRNA-based modulation of KMT5C-H4K20me3 as an anti-cancer approach would be particularly exciting in the current era of RNA therapeutics.

Additional vulnerabilities may stem from somatic mutations or germline SNPs within *KMT5C*. Several somatic mutations of *KMT5C* have been identified in cancer patients, yet their effects on activity or stability remain poorly understood (Figure 3B). Notably, one such mutation in *KMT5C* corresponds to a residue whose alteration in *KMT5B* abolishes methyltransferase activity [50], emphasizing the need to assess functional consequences of these variants. Indeed, these germline SNPs within *KMT5C* may influence its enzymatic activity, stability, and ability to form functional complexes with its interacting partners. Understanding these variants could inform strategies to revert deleterious mutations, including the use of AAV-mediated genome-editing [72].

Absence of a KMT5C-specific inhibitor or degrader remains a major obstacle for evaluating KMT5C as a therapeutic target (Figure 3C). Development of selective degraders, such as proteolysis-targeting chimeras (PROTACs), would enable both therapeutic intervention in tumors with KMT5C overexpression and mechanistic dissection of its role in regulating H4K20me3. While no KMT5C-specific compound exists, the dual KMT5B/C inhibitor A-196 has shown promise in preclinical models [73]. In HCC, where KMT5C is upregulated under stress, including after PARP inhibitor treatment, A-196 synergized with the PARP inhibitor to reduce tumor burden [64]. Similarly, in lung cancer with elevated KMT5C-H4K20me3, genetic ablation of KMT5C or pharmacological inhibition with A-196 enhanced anti-PD-L1 immunotherapy in immunocompetent mouse models [68]. A-196 also promotes CD4^+^ T cell differentiation, further supporting its potential synergy with immunotherapy [74]. Together, these preclinical findings highlight the therapeutic promise of targeting KMT5C-H4K20me3 in combination regimens.

Given the tissue- and context-specific roles of KMT5C-H4K20me3, accurate quantification at diagnosis will be critical. Beyond direct tumor biopsies, non-invasive methods may prove feasible. For example, nucleosomes carrying histone PTMs are released into bloodstream with circulating cell-free DNA and can be quantified by mass spectrometry or antibody-based assays such as enzyme-linked immunosorbent assay (ELISA) [75]. Notably, reduced H4K20me3 levels at repetitive elements have been detected in cell-free circulating nucleosomes from colorectal cancer patients, supporting their potential as novel biomarkers [76,77]. Similarly, nucleosomes packaged within extracellular vesicles secreted by tumor cells may provide another window into KMT5C-H4K20me3 status [78]. However, whether these circulating nucleosomes or vesicle-associated nucleosomes reliably reflect the chromatin landscape of cancer cells remains to be determined.

## Conclusions

Although traditionally viewed as a tumor suppressor, emerging evidence suggests that KMT5C-mediated H4K20me3 may also exhibit oncogenic functions in a context-dependent manner, underscoring the need to reevaluate its role across cancer types. This complexity highlights that the function of KMT5C-H4K20me3 extends beyond its established role in heterochromatin formation. Indeed, several studies suggest that KMT5C can also be recruited to euchromatic regions through non-canonical, HP1-independent mechanisms, where it deposits H4K20me3 and represses transcription of genes outside of constitutive heterochromatin. These findings point to the importance of identifying KMT5C’s binding partners beyond HP1 and characterizing its gene targets in euchromatic regions.

However, key technical and translational challenges remain. Currently, no small molecule inhibitors specifically target KMT5C. The dual KMT5B/C inhibitor A-196 has been used to probe KMT5C function [24,62,73], but its therapeutic relevance is uncertain. Notably, KMT5B/C double knockout mice exhibit prenatal lethality, though this finding is based on conditional models rather than temporally or spatially controlled deletions [23]. In contrast, KMT5C-null mice are viable and develop normally with no apparent phenotypic abnormalities [23], suggesting that selective KMT5C inhibition may be well tolerated, strengthening its candidacy as a therapeutic target. Nonetheless, the absence of a KMT5C-specific inhibitor limits efforts to directly evaluate its therapeutic potential. The development of KMT5C-specific degraders, such as PROTACs or auxin-inducible systems, will be essential to move this field forward.

Another barrier is the lack of high-quality, reproducible antibodies to measure endogenous KMT5C protein levels (unpublished data). Generating these tools, along with defining the function of KMT5C-H4K20me3, will be critical steps towards validating KMT5C as a therapeutic target.

Evidence also suggests that H4K20me3 is dynamically regulated through reversible demethylation, although the enzymes responsible remain uncertain. Several candidate demethylases have been proposed: hHR23A and hHR23B, homologs of yeast RAD23, demethylate all three H4K20 methylation states *in vitro*, and their overexpression reduces global H4K20 methylation in cells [79]. PHF2 has also been implicated in demethylating H4K20me3 deposited by SMYD5, acting as part of the nuclear receptor corepressor 1 (NCoR) complex in a p65-dependent manner in *Drosophila* Schneider cells [35]. Whether these findings extend to mammalian systems, and their contribution to the dynamic regulation of H4K20me3 in normal and cancer contexts, requires further investigations. Such insights could position H4K20me3 demethylases as an additional therapeutic target.

In addition to its canonical role in methylating histone substrates, KMT5C may also act on yet-to-be identified non-histone substrates that contribute to the phenotypes observed in cancers with dysregulated KMT5C. For instance, the lysine methyltransferase SMYD5 was recently shown to methylate core ribosomal protein L40 (rpL40), thereby altering mRNA translation and driving oncogenesis in gastric adenocarcinoma [37]. This raises the possibility that KMT5C and other lysine methyltransferases regulate oncogenic pathways through modification of non-histone substrates, an area requiring further study.

Beyond cancer, KMT5C-H4K20me3 has also been implicated in other diseases. KMT5C regulates gluconeogenesis-related genes in hepatocytes independent of its methyltransferase activity [80], controls thermogenic-related gene expression in adipocytes through H4K20me3 [27], and H4K20me3 levels are reduced in murine models of heart disease [81]. These findings expand the relevance of KMT5C-H4K20me3 to metabolic and cardiovascular disease, further underscoring its therapeutic potential.

Taken together, dysregulation of KMT5C-H4K20me3 across multiple cancers and other diseases positions it as an attractive yet complex therapeutic target. Realizing this potential will require resolving the context-dependent variability in KMT5C-H4K20me3 function, elucidating the molecular mechanisms governing its recruitment and enzymatic activity, and developing selective pharmacological tools. Ultimately, these efforts will determine whether KMT5C-H4K20me3 can be leveraged safely and effectively as a therapeutic target.

## Data Availability

No datasets were generated or analyzed during the current study.

## List of abbreviations

PTM: Post-Translational Modification
LUSC: Lung Squamous cell Carcinoma
pHGG: Pediatric High-Grade glioma
HDAC: Histone deacetylase
FDA: Food and Drug Administration
EZH2: Enhancer of Zeste Homolog 2
AAV: Adeno-Associated Virus
KMT: Lysine Methyltransferase
MEF: Mouse Embryonic Fibroblast
FRAP: Fluorescence After Photobleaching
rDNA: Ribosomal DNA
EGFR: Epidermal Growth Factor Receptor
TKI: Tyrosine kinase inhibitor
NSCLC: Non-small cell lung cancer
ER: Estrogen Receptor
DDR: DNA damage repair
HCC: Hepatocellular carcinoma
AURKB: Aurora Kinase B
HP1: Heterochromatin protein 1
NCoR: Nuclear Receptor Corepressor 1
miRNA: microRNA
PROTAC: Proteolysis-targeting chimera
SNP: Single Nucleotide Polymorphisms
